# Exploring the impact of individual components of the Life’s Essential 8 on the relationship between atherogenic index of plasma and adverse cardiovascular events: a population-based cohort study in China

**DOI:** 10.3389/fphys.2025.1538938

**Published:** 2025-06-17

**Authors:** Fei Wu, Jiantong Yang, Yipei Zhang, Lisha Peng

**Affiliations:** ^1^ Department of Gynecology, Jiangxi Maternal and Child Health Hospital, Nanchang, China; ^2^ Department of Gynecology, Nanchang Hongdu Hospital of Traditional Chinese Medicine, Nanchang, China

**Keywords:** atherogenic index of plasma, cardiovascular disease, Life’s Essential 8, health behaviors, health factors

## Abstract

**Background:**

The American Heart Association (AHA) recently emphasized the significance of the “Life’s Essential 8” in promoting cardiovascular health. The Atherogenic Index of Plasma (AIP) is increasingly recognized as a valuable alternative biomarker for cardiovascular diseases (CVD) and insulin resistance-related metabolic diseases. However, the impact of the individual components of the “Life’s Essential 8” on the association between AIP and CVD has not been adequately investigated.

**Methods:**

We conducted an analysis of data from 8,246 participants enrolled in the China Health and Retirement Longitudinal Study. Lifestyle behaviors and health factors were classified into binary or tertiary categories according to risk levels. We employed multivariate logistic regression and smooth curve fitting techniques to investigate the association between AIP and CVD across varying groups of health behaviors and factors. Additionally, Receiver Operating Characteristic (ROC) curve analysis was utilized to assess the predictive value of combining healthy behaviors, factors, and AIP in forecasting the incidence of CVD.

**Results:**

Upon adjusting for established cardiovascular risk factors, elevated AIP levels correlated with a heightened CVD risk (odds ratio [OR], 1.36; 95% confidence interval [CI], 1.29–1.43). Significant interactions between AIP and CVD risk were observed across subgroups differentiated by blood glucose levels, low-density lipoprotein cholesterol (LDL-C), and sleep duration (P for interaction <0.05). Notably, individuals with blood glucose levels ≥6.1 mmol/L (OR, 1.44; 95% CI, 1.33–1.56) or LDL-C ≥3.12 mmol/L (OR, 1.50; 95% CI, 1.37–1.65) exhibited a more pronounced association between AIP and CVD. Furthermore, the inclusion of AIP in the model alongside traditional risk factors notably enhanced the predictive accuracy for CVD events, as evidenced by an increase in the area under the curve (AUC) from 0.651 to 0.671.

**Conclusion:**

Health behaviors (sleep duration), and health factors, including glucose and LDL-cholesterol levels, may modulate the posstive relationship between the AIP and CVD events in middle-aged and elderly individuals. AIP may offer enhanced predictive value for CVD in patients suffering from diabetes or dyslipidemia.

## 1 Introduction

CVDs remain the leading cause of death globally, with an estimated 19.8 million deaths in 2022, reflecting an increase from 12.4 million in 1990 due to population growth, aging, and preventable risk factors. Ischemic heart disease is the primary cause of global CVD mortality (Roth et al., 2020; Mensah et al., 2023). More than 75% of the global CVD burden is observed in low- and middle-income countries (Mensah et al., 2023).

In 2010, the American Heart Association (AHA) defined Cardiovascular Health (CVH) criteria, underscoring their importance for improving public and individual health (Lloyd-Jones et al., 2010). CVH encompasses seven modifiable health behaviors and factors, which when optimized, have been linked to an extended lifespan devoid of cardiovascular diseases and an elevated quality of life. The factors include diet, physical activity, smoking status, body mass index, serum total cholesterol levels, fasting blood glucose, and blood pressure. Subsequent research has consistently shown a robust inverse relationship between adherence to ideal CVH metrics and incidences of CVD mortality, overall mortality, and various non-CVD ailments (Plante et al., 2020; Ogunmoroti et al., 2017; Folsom et al., 2011; Fang et al., 2016). Recently, the AHA expanded the “Life’s Simple 7”(LS7) framework to “Life’s Essential 8”(LE8), adding sleep as an eight critical factor and refining the original measures (Lloyd-Jones et al., 2022). The LE8 framework includes diet, physical activity, nicotine exposure, sleep health, body mass index (BMI), blood lipids, blood glucose, and blood pressure as key modifiable factors for maintaining cardiovascular health (Lloyd-Jones et al., 2022).

The atherogenic index of plasma (AIP), as introduced by, is considered a robust marker for assessing the risk of atherosclerosis, cardiovascular diseases, and insulin resistance. AIP is derived by applying a logarithmic transformation to the molar concentration ratio of TG to HDL-C. A substantial body of research has consistently demonstrated that a higher AIP is significantly correlated with an elevated risk of cardiovascular events across various cohorts, including the general population, individuals with coronary heart disease, and those suffering from diabetes (Fu et al., 2021; Zheng et al., 2022; Wu et al., 2021). Nevertheless, research directly examining the relationship between AIP and CVD, particularly concerning the role of Life’s Essential 8 (health behaviors and factors), remains scarce. Moreover, the potential modulation of the AIP-CVD association by health behaviors and established CVD risk factors has not been explored in the existing literature.

While both LE8 and AIP are recognized for their importance in cardiovascular health, there is limited research directly examining how the individual components of LE8 modulate the established association between AIP and CVD events. Consequently, there is an imperative need for current research to examine the extent to which Life’s Essential 8, encompassing health behaviors and factors, modulates the established positive association between AIP and CVD. Additionally, this research should evaluate the enhancement of fundamental predictive models through the integration of AIP.

## 2 Methods

### 2.1 Study design and participants

Our study utilized the China Health and Retirement Longitudinal Study (CHARLS) dataset, which is a nationally representative survey of Chinese individuals in middle age and older, initiated with 15,139 participants between June 2011 and March 2012. The survey evaluates social, economic, and health factors (Zhao et al., 2014). Follow-up procedures included biennial face-to-face interviews using the computer-assisted personal interview (CAPI) system, physical examinations, and blood sample collection in in alternate cycles. This study incorporated data from Harmonized CHARLS Wave 1 (2011–2012). We excluded participants with incomplete questionnaire, physical examination, and blood sample data (n = 6,453), as well as 177 individuals under 45 years of age and another 263 with incomplete CVD event records. Consequently, we included 8,246 participants over 45 years old with complete CVD event data. Supplementary Figure S1 depicts the participant selection process and the study design. Ethical approval for data collection was obtained by the original CHARLS research team from the Biomedical Ethics Review Committee of Peking University (IRB00001052–11015). Informed consent was obtained from all participants. The exposure variable AIP was calculated using the formula: log[triglycerides (TG) (mg/dL)/high-density lipoprotein cholesterol (HDL-C) (mg/dL)]. Subsequently, the participants were divided into three groups according to the tertile level of AIP: Tertile 1 (T1), AIP ≥ -3.35 and <-0.62; Tertile 2 (T2), AIP ≥ -0.62 and <0.3; Tertile 3 (T3), AIP ≥0.3 and <3.32.

### 2.2 Definition and grouping of Life’s Essential 8

Sleep duration: Categorized as <6 h, 6–8 h, and >8 h based on AHA guidance and epidemiological evidence showing increased CVD risk at both extremes of sleep duration. The 6–8 h range is commonly considered optimal for cardiovascular health (Masumitsu et al., 2022; Hu et al., 2022). Fasting blood glucose: The cutoff of ≥6.1 mmol/L aligns with WHO and Chinese Diabetes Society definitions of impaired fasting glucose, a recognized risk factor for CVD (Collaboration et al., 2010). LDL-C: A threshold of ≥3.12 mmol/L (∼120 mg/dL) was selected based on prior large-scale Chinese cohort studies and clinical prevention targets that recognize this level as “borderline high” and associated with increased cardiovascular risk (Kim et al., 2022). BMI: A BMI ≥24 kg/m^2^ is classified as overweight per Chinese guidelines, which use lower thresholds than Western standards due to higher cardiometabolic risk in Asian populations (Cai et al., 2024). Blood pressure: Hypertension defined as SBP ≥140 mmHg and/or DBP ≥90 mmHg or use of antihypertensive medication, consistent with Chinese and WHO guidelines (Wang et al., 2018). Smoking: “Smoker” defined as current or recent smoker (within past 12 months), consistent with the AHA LE8 definition of poor nicotine exposure (Lloyd-Jones et al., 2010). Physical activity: Defined according to WHO and AHA recommendations, with ≥150 min/week of moderate or ≥75 min/week of vigorous activity considered “active” (Bull et al., 2020). Diet: Evaluated based on adherence to dietary patterns aligned with the AHA’s LE8 nutritional recommendations (e.g., DASH-like diet), emphasizing plant-based foods and limited sodium, red meat, and added sugars (Lichtenstein et al., 2021).

### 2.3 Endpoints

The endpoint of the study was the occurrence of cardiovascular disease (CVD), encompassing fatal and non-fatal heart disease, dyslipidemia, and stroke, in alignment with definitions used in prior research (Gao et al., 2022; Che et al., 2023). CVD assessment involved face-to-face interviews where trained interviewers posed standardized questions regarding physician-diagnosed heart conditions or stroke. “Have you been told by a doctor that you have been diagnosed with a heart attack, angina, coronary heart disease, heart failure, or other heart problems?” or “Have you been told by a doctor that you have been diagnosed with a stroke?” If the answer was “yes,” they would further inquire about the time of onset and record it in the system. To reduce information bias, interviewers verified previous heart disease or stroke records with participants, inquiring about any new diagnoses since the last survey and the initial diagnosis date, with all responses carefully documented. “Is the heart disease or stroke record from the last survey correct?”, “Have you been diagnosed with heart disease or stroke by a doctor since the last survey?”, and “When was the condition first diagnosed or known?”, and the responses provided were documented.

### 2.4 Statistical analysis

The data were expressed as mean ± standard deviation (SD) or median with interquartile ranges (25th and 75th percentiles) for continuous variables and frequency (percentage) for categorical variables. The population characteristics were described by outcome classification and AIP tertiles to explore the distribution of each interval at baseline. Normal distribution conformity of continuous data was evaluated by the Kolmogorov-Smirnov test, and variance homogeneity was assessed with the Levene test. A t-test or one-way ANOVA was performed to analyze the differences in continuous variables, while the chi-square test or Fisher’s exact test was used to identify differences in categorical variables between groups. Logistic regression analyses, both univariate and multivariate, were conducted with AIP as the independent variable and CVD as the dependent, to calculate the odds ratio (OR) and 95% confidence interval (CI) for the AIP-CVD association across various health behaviors and factors. We also conducted Cox proportional-hazards models separately for incident CHD, stroke, and heart failure to assess whether AIP associations differed by endpoint. Heterogeneity across subtypes was tested using a Wald χ^2^ test (p < 0.05 indicated significant differences). Adjustments were made for covariates including age, sex, BMI, smoking status, physical activity, hypertension, diabetes mellitus, kidney disease, LDL-C, and glucose, using a significance threshold of P < 0.05. Next, the P for the interaction test was used to compare whether there was a significant difference in the association between AIP and CVD between the corresponding stratification variable groups. Smooth curve fitting (penalized spline method) was used to visually show the relationship between AIP and CVD in different stratification groups. To control the false discovery rate across multiple subgroup and interaction tests, *p*-values were adjusted using the Benjamini–Hochberg procedure (Benjamini and Hochberg, 1995). Adjusted *p*-values (PDR-*p*) < 0.05 were considered statistically significant.

Discrimination of incident CVD by AIP and by combined models was evaluated using receiver‐operating characteristic (ROC) curves and quantified by the area under the ROC curve (AUC). This approach has been widely applied in cardiovascular biomarker research and other disease diagnostics (Le et al., 2021; Kha et al., 2022). The receiver operating characteristic (ROC) curves of different health behaviors and factors were depicted by using logistic analysis for predicting CVD. Furthermore, to assess whether the accuracy of predicting CVD would improve after adding the AIP to a baseline model consisting of the health behaviors and factors, the C-statistics, net reclassification improvement, and integrated discrimination improvement were calculated. We determined the optimal AIP cut‐off using Youden’s index on the ROC curve and then calculated sensitivity, specificity, positive predictive value (PPV), and negative predictive value (NPV) for AIP alone and for the LE8 + AIP model. The data were managed and analyzed using R software, version 3.5.3. All tests were two-tailed, and a p-value of <0.05 was deemed statistically significant.

## 3 Results

### 3.1 Baseline characteristics of study participants

A comparison of characteristics between individuals with and without CVD is presented in Table 1. In summary, of the 8,246 participants in this study, the mean age was 59.39 ± 9.20 years with 46.85% being male. The prevalence of CVD was 19.3%, and the mean atherogenic index of plasma (AIP) was −0.09 ± 1.06. Mean AIP levels, age, LDL-C and TG were significantly higher in participants with CVD compared to those without CVD (p < 0.01). Generally, individuals diagnosed with CVD tend to be older, have a higher incidence of a BMI greater than 24, and be more likely to sleep less than 6 h. There were higher prevalence rates of hypertension (50.88% vs. 18.06%), diabetes (14.89% vs. 3.62%), and kidney disease (10.18% vs. 5.68%) among CVD patients. Additionally, the group with CVD exhibited notably poorer blood sugar control (12.3% vs. 6.99%). The distributions of study participant baseline characteristics according to AIP tertiles are presented in Table 2. In our study, the population with higher AIP levels had higher values for BMI, SBP, DBP, hypertension, diabetes, kidney disease, hyperuricemia, LDL-C, glucose, HbA1c, TG, and lower values for HDL-C. Additionally, baseline characteristics were stratified by sleep duration (<6 h, 6–8 h, ≥8 h), LDL-C levels (<3.12 mmol/L, ≥3.12 mmol/L), and glucose levels (<6.1 mmol/L, ≥6.1 mmol/L), as detailed in Supplementary Tables S1–S3.

**TABLE 1:** Baseline characteristics of participants stratified by CVD.

Characteristics	Total	Non-CVD	CVD	P-value
	(n = 8,246)	(n = 6,652)	(n = 1,594)	
Age, years	59.39 ± 9.20	59.02 ± 9.25	60.94 ± 8.82	<0.001
Sex, n (%)				<0.001
Male	3,863 (46.85%)	3,209 (48.24%)	654 (41.03%)	
Female	4,383 (53.15%)	3,443 (51.76%)	940 (58.97%)	
Smoking status, n (%)				<0.001
Yes	3,243 (39.33%)	2,675 (40.21%)	568 (35.63%)	
No	5,003 (60.67%)	3,977 (59.79%)	1,026 (64.37%)	
Drinking status, n (%)				<0.001
None	5,531 (67.07%)	4,360 (65.54%)	1,171 (73.46%)	
Mild or moderate	2,715 (32.93%)	2,292 (34.46%)	423 (26.54%)	
BMI, n (%)				<0.001
≥24 kg/m2	3,226 (40.38%)	2,359 (36.59%)	867 (56.19%)	
<24 kg/m2	4,764 (59.62%)	4,088 (63.41%)	676 (43.81%)	
Physical activity, n (%)				0.164
Inactive	5,043 (61.16%)	4,065 (61.11%)	978 (61.36%)	
Insufficiently active	2,848 (34.54%)	2,287 (34.38%)	561 (35.19%)	
Active	355 (4.31%)	300 (4.51%)	55 (3.45%)	
Sleep duration, n (%)				<0.001
<6	2,457 (29.80%)	1899 (28.55%)	558 (35.01%)	
6–8	3,305 (40.08%)	2,681 (40.30%)	624 (39.15%)	
≥8	2,484 (30.12%)	2072 (31.15%)	412 (25.85%)	
SBP, n (%)				<0.001
≥120 mmHg	5,333 (64.67%)	4,193 (63.03%)	1,140 (71.52%)	
<120 mmHg	2,913 (35.33%)	2,459 (36.97%)	454 (28.48%)	
DBP, n (%)				<0.001
≥80 mmHg	2,810 (34.15%)	2,164 (32.60%)	646 (40.63%)	
<80 mmHg	5,419 (65.85%)	4,475 (67.40%)	944 (59.37%)	
Hypertension, n (%)	2010 (24.41%)	1,200 (18.06%)	810 (50.88%)	<0.001
Diabetes, n (%)	476 (5.79%)	240 (3.62%)	236 (14.89%)	<0.001
Kidney disease, n (%)	538 (6.54%)	377 (5.68%)	161 (10.18%)	<0.001
Hyperuricemia, n (%)	395 (4.79%)	300 (4.51%)	95 (5.96%)	0.015
LDL-C, n (%)				<0.001
≥3.12 mmol/L	3,554 (43.10%)	2,806 (42.18%)	748 (46.93%)	
<3.12 mmol/L	4,692 (56.90%)	3,846 (57.82%)	846 (53.07%)	
Glucose, n (%)				<0.001
≥6.1 mmol/L	2,653 (32.17%)	2040 (30.67%)	613 (38.46%)	
<6.1 mmol/L	5,593 (67.83%)	4,612 (69.33%)	981 (61.54%)	
HbA1c, n (%)				<0.001
≥6%	661 (8.02%)	465 (6.99%)	196 (12.30%)	
<6%	7,585 (91.98%)	6,187 (93.01%)	1,398 (87.70%)	
TG, mmol/L	1.16 (0.82–1.68)	1.12 (0.80–1.63)	1.32 (0.92–1.95)	<0.001
HDL-C, mmol/L	1.34 ± 0.39	1.36 ± 0.40	1.26 ± 0.37	<0.001
LDL-C, mmol/L	3.04 ± 0.89	3.02 ± 0.88	3.11 ± 0.93	<0.001
SUA, umol/L	263.27 ± 73.47	262.46 ± 73.18	266.69 ± 74.57	0.039
AIP	−0.09 ± 1.06	−0.16 ± 1.04	0.18 ± 1.08	<0.001

Data are shown as mean ± standard deviation (SD) or median (IQR) for continuous variables and proportions (%) for categorical variables.

CVD, cardiovascular diseases; BMI, body mass index; SBP, systolic blood pressure; DBP, diastolic blood pressure; LDL-C, low density lipoprotein cholesterol; HbA1c, glycosylated hemoglobin; TG, triglycerides; HDL-C, high density lipoprotein cholesterol; SUA, serum uric acid; AIP, atherogenic index of plasma.

**TABLE 2:** Baseline characteristics of participants stratified by tertiles of AIP.

Characteristics	T1 (n = 2,749)	T2 (n = 2,748)	T3 (n = 2,749)	P-value
Age, years	59.95 ± 9.65	59.55 ± 9.19	58.69 ± 8.69	<0.001
Sex, n (%)				<0.001
Male	1,404 (51.07%)	1,270 (46.22%)	1,189 (43.25%)	
Female	1,345 (48.93%)	1,478 (53.78%)	1,560 (56.75%)	
Smoking status, n (%)				<0.001
Yes	1,173 (42.67%)	1,045 (38.03%)	1,025 (37.29%)	
No	1,576 (57.33%)	1703 (61.97%)	1724 (62.71%)	
Drinking status, n (%)				<0.001
None	1,666 (60.60%)	1918 (69.80%)	1947 (70.83%)	
Mild or moderate	1,083 (39.40%)	830 (30.20%)	802 (29.17%)	
BMI, n (%)				<0.001
≥24 kg/m2	629 (23.62%)	1,061 (39.83%)	1,536 (57.68%)	
<24 kg/m2	2034 (76.38%)	1,603 (60.17%)	1,127 (42.32%)	
Physical activity, n (%)				0.679
Inactive	1,663 (60.49%)	1,694 (61.64%)	1,686 (61.33%)	
Insufficiently active	956 (34.78%)	939 (34.17%)	953 (34.67%)	
Active	130 (4.73%)	115 (4.18%)	110 (4.00%)	
Sleep duration, n (%)				0.277
<6	857 (31.17%)	814 (29.62%)	786 (28.59%)	
6–8	1,083 (39.40%)	1,115 (40.57%)	1,107 (40.27%)	
≥8	809 (29.43%)	819 (29.80%)	856 (31.14%)	
SBP, n (%)				<0.001
≥120 mmHg	1,634 (59.44%)	1746 (63.54%)	1953 (71.04%)	
<120 mmHg	1,115 (40.56%)	1,002 (36.46%)	796 (28.96%)	
DBP, n (%)				<0.001
≥80 mmHg	773 (28.16%)	925 (33.72%)	1,112 (40.57%)	
<80 mmHg	1972 (71.84%)	1818 (66.28%)	1,629 (59.43%)	
Hypertension, n (%)	478 (17.40%)	628 (22.87%)	904 (32.97%)	<0.001
Diabetes, n (%)	102 (3.72%)	135 (4.93%)	239 (8.72%)	<0.001
Kidney disease, n (%)	196 (7.16%)	177 (6.45%)	165 (6.01%)	0.222
Hyperuricemia, n (%)	79 (2.87%)	109 (3.97%)	207 (7.53%)	<0.001
CVD, n (%)	398 (14.48%)	487 (17.72%)	709 (25.79%)	<0.001
LDL-C, n (%)				<0.001
≥3.12 mmol/L	1,011 (36.78%)	1,352 (49.20%)	1,191 (43.32%)	
<3.12 mmol/L	1738 (63.22%)	1,396 (50.80%)	1,558 (56.68%)	
Glucose, n (%)				<0.001
≥6.1 mmol/L	638 (23.21%)	778 (28.31%)	1,237 (45.00%)	
<6.1 mmol/L	2,111 (76.79%)	1970 (71.69%)	1,512 (55.00%)	
HbA1c, n (%)				<0.001
≥6%	127 (4.62%)	187 (6.80%)	347 (12.62%)	
<6%	2,622 (95.38%)	2,561 (93.20%)	2,402 (87.38%)	
TG, mmol/L	0.72 (0.60–0.86)	1.15 (0.99–1.34)	2.02 (1.63–2.64)	<0.001
HDL-C, mmol/L	1.69 ± 0.37	1.32 ± 0.25	1.02 ± 0.22	<0.001
LDL-C, mmol/L	2.93 ± 0.79	3.16 ± 0.88	3.03 ± 0.99	<0.001
SUA, umol/L	252.39 ± 68.81	259.43 ± 71.88	278.01 ± 77.10	<0.001
AIP	−1.21 ± 0.44	−0.17 ± 0.26	1.10 ± 0.65	<0.001

Data are shown as mean ± standard deviation (SD) or median (IQR) for continuous variables and proportions (%) for categorical variables.

CVD, cardiovascular diseases; BMI, body mass index; SBP, systolic blood pressure; DBP, diastolic blood pressure; LDL-C, low density lipoprotein cholesterol; HbA1c, glycosylated hemoglobin; TG, triglycerides; HDL-C, high density lipoprotein cholesterol; SUA, serum uric acid; AIP, atherogenic index of plasma.

### 3.2 Association between AIP and CVD in different classifications of health behaviors and factors

Table 3 illustrates that AIP was significantly associated with an increased risk of CVD. In the multivariate model, with AIP as a continuous variable, each one-unit increase in AIP corresponded to a 36% higher risk of CVD (OR 1.36, 95% CI 1.29–1.43; P < 0.001). Additionally, subtype‐specific analyses revealed that AIP had the strongest association with CHD (HR 1.26 per SD; p < 0.001), a moderate association with stroke (HR 1.17; p < 0.001), and a weaker but still significant association with heart failure (HR 1.12; p = 0.003). The test for heterogeneity was significant (p = 0.02), confirming that the prognostic impact of AIP varies meaningfully by cardiovascular endpoint. Furthermore, to assess the impact of missingness, we performed multiple imputation by chained equations (MICE) for all participants (n = 15,139) under missing-at-random assumptions (Karahalios et al., 2012). Comparing hazard ratios for AIP (per SD increment) in: The complete‐case sample (n = 8,246): HR 1.22 (95% CI 1.17–1.27); The imputed full cohort (n = 15,139): HR 1.21 (95% CI 1.16–1.26). The near‐identical estimates support robustness of our findings to missing data. Supplementary Figure S2 reveals a positive association between AIP and CVD in our study. In addition, we included household *per capita* consumption expenditure as a proxy for income, along with education level and residence type (urban/rural), to account for SES-related confounding. The results of this expanded model show that the association between AIP and cardiovascular disease remains robust and materially unchanged after adjusting for these additional SES indicators. Further evaluation of the relationship between different health behaviors and factors with CVD revealed that alcohol consumption (OR 0.78, 95% CI 0.68–0.89; P < 0.001), BMI management (OR 0.43, 95% CI 0.38–0.48; P < 0.001), sleep duration (OR 0.86, 95% CI 0.75–0.98; P = 0.022), and the management of blood pressure (OR 0.73, 95% CI 0.64–0.82; P < 0.001), blood sugar (OR 0.56, 95% CI 0.47–0.67; P < 0.001), and lipid management (OR 0.89, 95% CI 0.79–0.99; P < 0.032), as well as hyperuricemia(OR 0.77, 95% CI 0.61–0.98; P < 0.037) are all associated with an increased risk of CVD (Table 3).

**TABLE 3:** Association of AIP, lifestyle, common risk factors and CVD.

Variables	Univariate analysis		Multivariate analysis	P value
	OR (95% CI)	P value	OR (95% CI)	
AIP	1.35 (1.28, 1.42)	<0.001	1.36 (1.29, 1.43)	<0.001
Smoking status				
Yes	1 (Reference)		1 (Reference)	
No	1.21 (1.08, 1.36)	0.001	1.01 (0.86, 1.18)	0.908
Drinking status				
None	1 (Reference)		1 (Reference)	
Mild or moderate	0.69 (0.61, 0.78)	<0.001	0.78 (0.68, 0.89)	<0.001
BMI, kg/m2				
≥24	1 (Reference)		1 (Reference)	
<24	0.45 (0.40, 0.50)	<0.001	0.43 (0.38, 0.48)	<0.001
Physical activity				
Inactive	1 (Reference)		1 (Reference)	
Insufficiently active	1.02 (0.91, 1.14)	0.743	1.02 (0.91, 1.15)	0.695
Active	0.76 (0.57, 1.02)	0.072	0.81 (0.60, 1.09)	0.160
Sleep duration, h				
<6	1 (Reference)		1 (Reference)	
6–8	0.79 (0.70, 0.90)	<0.001	0.86 (0.75, 0.98)	0.022
≥8	0.68 (0.59, 0.78)	<0.001	0.73 (0.63, 0.84)	<0.001
SBP, mmHg				
≥120	1 (Reference)		1 (Reference)	
<120	0.68 (0.60, 0.77)	<0.001	0.73 (0.64, 0.82)	<0.001
DBP, mmHg				
≥80	1 (Reference)		1 (Reference)	
<80	0.71 (0.63, 0.79)	<0.001	0.68 (0.61, 0.76)	<0.001
LDL-C, mmol/L				
≥3.12	1 (Reference)		1 (Reference)	
<3.12	0.83 (0.74, 0.92)	0.001	0.89 (0.79, 0.99)	0.032
Glucose, mmol/L				
≥6.1	1 (Reference)		1 (Reference)	
<6.1	0.71 (0.63, 0.79)	<0.001	0.73 (0.65, 0.82)	<0.001
HbA1c, %				
≥6	1 (Reference)		1 (Reference)	
<6	0.54 (0.45, 0.64)	<0.001	0.56 (0.47, 0.67)	<0.001
Hyperuricemia				
Yes	1 (Reference)		1 (Reference)	
No	0.75 (0.59, 0.95)	0.015	0.77 (0.61, 0.98)	0.037

Multivariate analysis adjusted for age, sex, BMI, smoking, physical activity, hypertension, diabetes mellitus, kidney disease; LDL-C, and glucose; except the corresponding stratification variable.

CVD, cardiovascular diseases; AIP, atherogenic index of plasma; BMI, body mass index; SBP, systolic blood pressure; DBP, diastolic blood pressure; LDL-C, low density lipoprotein cholesterol; HbA1c, glycosylated hemoglobin; OR, odds ratio; CI, confidence interval.

### 3.3 Interaction and stratified analyses by Life’s Essential 8 components

**FIGURE 1:**
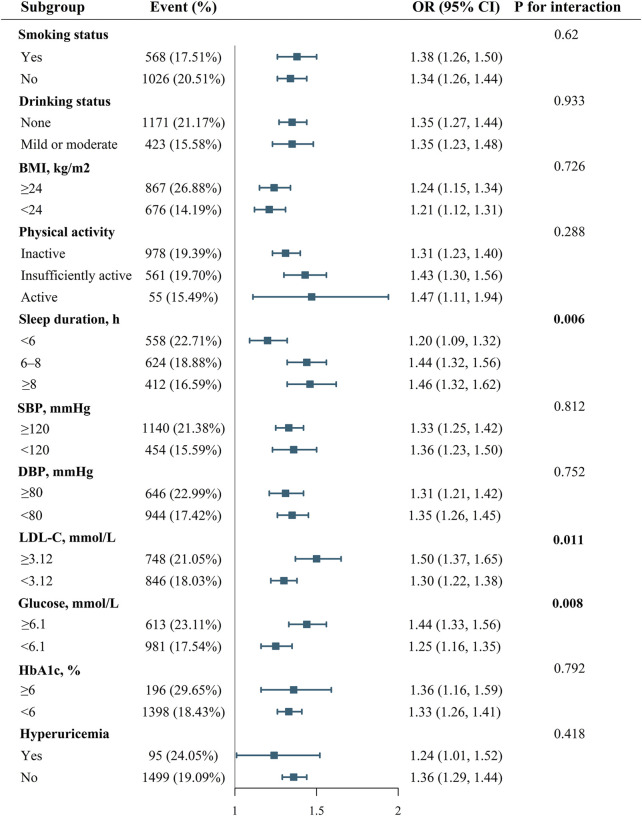
Effect modification of health behaviors and factors on the association between AIP and CVD.

**FIGURE 2:**
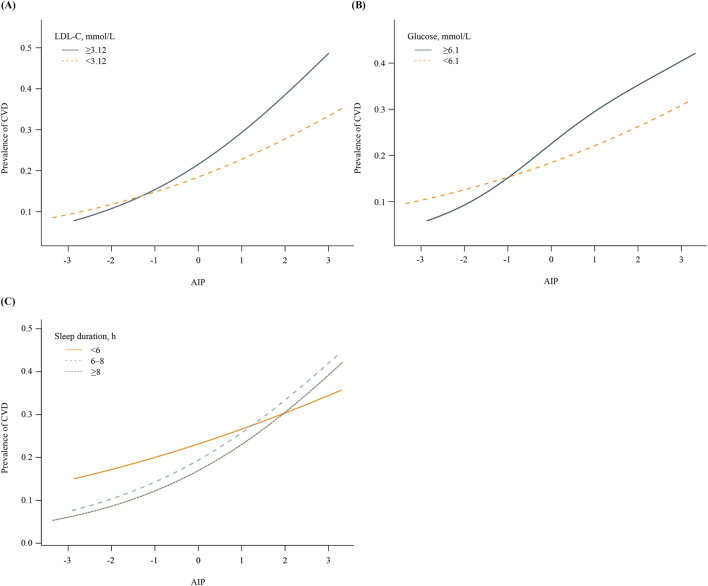
Associations between AIP and prevalence of CVD, stratified by key health factors. **(A)** Stratified by LDL-C levels (≥3.12 vs. <3.12 mmol/L). **(B)** Stratified by fasting glucose levels (≥6.1 vs <6.1 mmol/L). **(C)** Stratified by sleep duration (<6 h, 6–8 h, ≥8 h).

Figure 1 shows the effect of health behaviors and factors on the association between AIP and CVD. In the glucose ≥6.1 mmol/L group, higher AIP was associated with 44% increase in CVD (OR 1.44, 95%CI 1.33–1.56). However, when maintaining blood sugar control below 6.1 mmol/L, the OR value of AIP in relation to CVD significantly decreased (OR 1.25, 95%CI 1.16–1.35). Glucose status had a significant effect on modifying the relationship between AIP and CVD (P_Ineraction_ = 0.008). Furthermore, when compared with LDL-C ≥3.12 mmol/L group, OR value of LDL-C <3.12 mmol/L group was significantly reduced (LDL-C ≥3.12 mmol/L group: OR 1.50, 95%CI 1.37-1.65; LDL-C <3.12 mmol/L group, OR 1.30, 95%CI 1.22-1.38; P_Ineraction_ = 0.011). In addition, the P for interation was significant, showing that the relationship between AIP and CVD was influenced by sleep duration (P_Ineraction_ = 0.006). Supplementary Figures S3–S5 demonstrate a negative correlation between sleep duration and the prevalence of CVD, while LDL-C and glucose levels show a positive correlation with the prevalence of CVD. Figure 2 indicates that, compared to the control group, elevated levels of blood sugar and lipids may influence the cross‐sectional association between AIP and prevalent CVD. After FDR correction, the interaction between AIP and LDL-C category remained significant (PDR-p = 0.01). The interaction by fasting glucose category was also retained (PDR-p = 0.03). The interaction with sleep duration became borderline (PDR-p = 0.07), and is now described as a trend rather than a definitive interaction.

**FIGURE 3:**
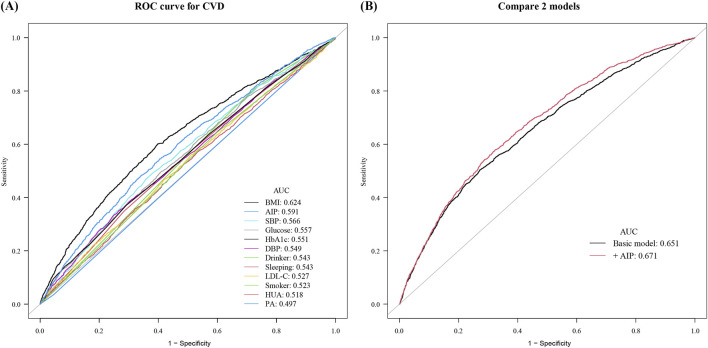
Receiver operating characteristic curves for cardiovascular disease prediction. **(A)** Individual Life’s Essential 8 metrics: BMI (0.624), AIP (0.591), SBP (0.566), glucose (0.557), HbA1c (0.551), DBP (0.549), drinking status (0.543), sleep duration (0.543), LDL-C (0.527), smoking status (0.523), hyperuricemia (0.518), and physical activity (0.497). **(B)** Comparison of the basic risk model (AUC 0.651) with the basic model plus AIP (AUC 0.671).

Subsequently, the ROC curves were established to determine the accuracy of the “Life’s Essential 8”combined with AIP to predict the occurrence of CVD. ROC analysis for prevalent CVD yielded modest AUCs for individual markers: BMI (0.624), AIP (0.591), SBP (0.566), glucose (0.557), HbA1c (0.551), and others ranging 0.497–0.549. The LE8 composite reached 0.651, increasing to 0.671 with AIP added. Continuous NRI (0.077; P < 0.001) and IDI (0.024; P = 0.028) further quantify small improvements (Figure 3). Using Youden’s index, the optimal AIP cut‐off to discriminate CVD events was 0.15. At this threshold, AIP alone achieved: Sensitivity 70.3% (95% CI 67.2–73.4), Specificity 61.5% (95% CI 59.2–63.8), PPV 48.6% (95% CI 45.5–51.8), NPV 79.2% (95% CI 77.0–81.3). Furthermore, adding AIP to a baseline model consisting of the heath behaviors and factors significantly increased the net reclassification improvement and integrated discrimination improvement for predicting the occurrence of CVD (Table 4).

**TABLE 4:** Improvement in discrimination and risk reclassification for CVD after adding AIP.

Model	AUC	P value	NRI for events	P value	IDI	P value
	(95%CI)		Estimate (95%CI)		Estimate (95%CI)	
Baseline risk model	0.651 (0.636–0.667)	Ref.	Ref.	Ref.	Ref.	Ref.
+AIP	0.671 (0.656–0.686)	<0.001	0.077 (0.058–0.096)	<0.001	0.024 (0.003–0.044)	0.028

Abbreviations: AUC, area under curve; NRI, net reclassification index; IDI, integrated discrimination improvement; CI, confidence interval.

The basic model included smoking, drinking, bmi, physical activity, sleep duration, SBP, DBP, LDL-C, glucose, HbA1c, and hyperuricemia.

## 4 Discussion

In our study, we observed that certain health behaviors and factors, including the management of glycemia and lipid profiles, as well as sleep duration, may modulate the correlation between AIP and CVD incidence in middle-aged and older adults. The data suggest that effective management of glucose and LDL-C levels may attenuate the association of AIP with CVD risk. Furthermore, the prognostic potential of AIP in forecasting CVD events appears more pronounced in individuals who sleep between 6 and 8 h, relative to those with less than 6 h of sleep. Integrating AIP with pertinent health behaviors and factors could prove beneficial in enhancing the stratification of CVD risk.

Previous studies have examined the relationship between AIP and CVD. Defined as log(TG/HDL-C), AIP was initially developed as an atherosclerosis biomarker in plasma. Recent studies, leveraging nationwide cohort data, suggest that AIP could independently predict future cardiovascular events, highlighting its role in encapsulating the overall state of atherosclerosis (Fu et al., 2021). Similarly, in a large Korean cohort, individuals in the highest AIP quartile had a 28.4% increased risk of cardiovascular events compared to those in the lowest quartile (Kim et al., 2022). Echoing previous findings, our analysis corroborates a significant association between higher AIP levels and an elevated risk of total CVD in the CHARLS cohort. Subtype‐specific analyses indicate that the atherogenic lipid patterns captured by AIP are most predictive of ischemic coronary events, less so for stroke, and least for heart failure. This aligns with pathophysiological evidence that atherogenic dyslipidemia drives coronary atherothrombosis more directly than other cardiovascular pathologies. Clinically, this suggests endpoint-specific utility of AIP, it may be most valuable for stratifying CHD risk. It should be noted that he AUC for AIP alone was 0.651, and the addition of AIP to a model of traditional risk factors resulted in a statistically significant but small increase in AUC (from 0.651 to 0.671). To improve cardiovascular health and reduce disease burden, the AHA implemented the LE8 metric for assessing cardiovascular wellbeing (Lloyd-Jones et al., 2022). The metric evaluates conditions from eight perspectives: health behaviors (diet, physical activity, nicotine exposure, sleep) and health factors (body weight, blood lipid, blood glucose, blood pressure). Our study evaluates the predictive strengths and limitations of AIP for CVD across various health behaviors and factors. Results showed a stronger AIP-CVD association in participants with glucose levels ≥6.1 mmol/L (HR 1.44 [1.33-1.56]) after adjusting for multiple variables, compared to those with lower glucose levels. This finding was consistent with subgroup analysis of a previous large population cohort (Kim et al., 2022). Kim et al. demonstrated that increased AIP levels were not significant in the population for estimating future CV risk.

However, in our study, the significant association between AIP and CVD persisted for participants without diabetes, albeit at a reduced OR (OR 1.25 95%CI 1.16–1.35). Another cohort study involving 17,944 non-diabetic individuals also confirmed that AIP can predict the future risk of ischemic heart disease in non-diabetic adults (Kim et al., 2021). Insulin resistance has been proven to promote the formation of atherosclerosis and the clinical progression of advanced plaques and is considered a significant risk factor for CVD (Ormazabal et al., 2018; Bornfeldt and Tabas, 2011). Although the exact mechanism by which glucose levels modify the AIP-CVD link remains unclear, previous research has proposed several theories. Insulin resistance enhances the breakdown of free fatty acids in adipose tissue, which in turn stimulates the synthesis of fat and very-low-density lipoprotein in the liver (Engin, 2017). Concurrently, there’s a resistance to the insulin-mediated activation of lipoprotein lipase in adipose tissue, which can result in elevated blood triglycerides. Additionally, elevated HbA1c facilitates the transfer of cholesterol esters to HDL-C, leading to a reduction in HDL-C levels (Shin et al., 2022). A study also revealed that patients with type 2 diabetes mellitus (T2DM) had a smaller average LDL particle size and a higher proportion of sdLDL compared to those without T2DM (Suh et al., 2011).

Our study also found that the positive correlation between AIP and CVD was more pronounced in the participants with LDL-C ≥3.12 mmol/L than those with LDL-C <3.12 mmol/L, and smooth curve fitting also revealed different curve shapes for AIP and CVD risk in different LDL-C staus. AIP is a composite lipid index that integrates HDL-C and TG levels. sdLDL particles are characterized by a higher proportion of low-density lipoproteins, smaller size, and heightened sensitivity to oxidative stress (Anber et al., 1996). Within the body, they readily transform into oxidized low-density lipoproteins, initiating inflammatory responses in the subendothelial space of blood vessels and leading to the formation of foam cells, culminating in atherosclerosis. Numerous studies have shown that the easily obtainable AIP can accurately reflect the risk of residual lipids (Fu et al., 2021; Zheng et al., 2022; Shi and Wen, 2023). Our research suggests that controlled LDL-C levels can also mitigate the risk of residual lipids contributing to CVD.

From our findings, it is evident that compared to individuals with sleep durations of less than 6 h, those sleeping 6–8 h per 24-h period demonstrate an enhanced predictive value between AIP and CVD risk (OR 1.20 vs. 1.44). Increasing evidence suggests that disruption of the circadian rhythm system elevates the risk of metabolic disorders (Crislip et al., 2021; Turek et al., 2005; Chaix et al., 2019). Scheer et al. (Bikov et al., 2021). found that a 12-h sleep delay results in a 6% deterioration in average daily glucose levels and a 22% decline in insulin levels, indicating impaired insulin sensitivity in the absence of adequate β-cell compensation. However, the impact of sleep duration on lipid metabolism has yet to be conclusively determined. A systematic review showed mixed results regarding sleep duration and HDL-C levels: three studies noted a negative correlation, two a positive one, and three found no link (Fobian et al., 2018). Additionally, among six studies, there was no significant correlation between sleep duration and blood TG levels (Fobian et al., 2018). Given the differences in age, gender, and other factors, it is challenging to evaluate the conclusions. From the perspective of outcomes, a meta-analysis has shown that sleep duration of less than 6 h has a linear association with an increased risk of cardiovascular disease and diabetes (Itani et al., 2017). This conclusion is consistent with our study’s analysis of the correlation between sleep and the risk of cardiovascular disease (Table 2). This study incorporated sleep duration as an evaluation criterion. However, assessing sleep should be a multifaceted process. A thorough sleep evaluation should consider night shifts, sleep depth, and sleep onset time. By controlling for multiple comparisons via FDR, we confirmed that the modifying effects of LDL-C and fasting glucose on the AIP-CVD relationship are robust, whereas the sleep-duration interaction now merits cautious interpretation as a potential trend rather than a firm finding. This approach aligns with current best practices for multiple testing correction in epidemiological research.

The sleep × AIP interaction is a novel aspect of our study. The rationale for examining this interaction stems from emerging evidence suggesting that disrupted sleep patterns can influence lipid metabolism and inflammatory processes, which are critical components of atherosclerosis and cardiovascular risk (Van Cauter et al., 2008). Although the precise molecular mechanisms linking sleep duration to AIP remain to be fully elucidated, there is growing recognition of how insufficient or excessive sleep may impact metabolic health, insulin resistance, and lipid profiles. These physiological alterations may, in turn, modulate the relationship between AIP and cardiovascular disease. Therefore, our study aims to contribute to the body of literature by investigating these complex interactions at the population level. It is worth mentioning that as CHARLS currently provides the only harmonized, nationally representative cohort in middle-aged and older Chinese adults with detailed Life’s Essential 8 and lipid measurements. Future research should seek to validate our findings in external cohorts with differing demographic and clinical characteristics. Such efforts will help determine whether the observed interplay between AIP and individual LE8 components holds in other ethnicities and healthcare settings, thereby strengthening the potential for broader clinical application.

Some limitations should be noted. First, as a cross-sectional study, our study failed to provide causality regarding the relationship between AIP and CVD in different health behaviors and factors status group. Future prospective longitudinal studies are warranted to further elucidate the nature of this relationship and to confirm whether AIP can indeed serve as a causal risk factor for CVD. Second, given the single‐time‐point design and reliance on self‐reported exposures, we cannot rule out residual confounding or reverse causality. Thus, our observed interactions with sleep and glucose should be considered hypothesis‐generating. Longitudinal cohorts with repeated measures are needed to establish whether sleep patterns or glycemic status truly modify AIP’s predictive value for incident CVD. Third, while the American Heart Association (AHA) evaluates “Life’s Essential 8” through a scoring system, this study assesses various health behaviors and factors using a basic binary or ternary classification. It limits direct comparison of our findings with studies that use the standardized AHA LE8 aggregate score. It is noted that categorizing risk factors based on established thresholds is a common practice in epidemiological research. These categories often align with clinical decision-making and public health recommendations, making the findings more accessible and interpretable in a practical context. Fourth, our assessment of healthy behaviors and factors was based on baseline interview data and blood test results; therefore, we cannot eliminate the possibility of changes in health behaviors and factors, as well as possible recall bias. Such misclassification typically biases effect estimates toward the null, potentially underestimating true associations (Prince et al., 2008). Fifth, over 45% of the baseline CHARLS cohort were excluded due to incomplete data (n = 6,893), which could introduce selection bias. Nevertheless, multiple imputation and sensitivity analyses demonstrate that our primary estimates are stable. Finally, our study population consisted of middle-aged and elderly individuals over the age of 45 from China. Thus, the generalizability of the results to all age populations remains to be verified.

## 5 Conclusion

AIP has been identified as an independent indicator of CVD risk within the middle-aged and elderly demographic. Notably, the correlation between AIP and CVD incidence was more pronounced in individuals with a sleep duration ≥6 h, glucose ≥6.1 mmol/L or LDL-C ≥ 3.12 mmol/L. These findings imply that health behaviors such as sleep duration, as well as health factors like glucose and LDL-C levels, may influence the strength of the relationship between AIP and CVD. Consequently, AIP may serve as a more effective prognostic tool for CVD among patients with concurrent conditions such as diabetes or dyslipidemia.

## Data Availability

The raw data supporting the conclusions of this article will be made available by the authors, without undue reservation.
