# Supplementary material for: Exploring the impact of individual components of the Life’s Essential 8 on the relationship between atherogenic index of plasma and adverse cardiovascular events: a population-based cohort study in China
Source: Front Physiol. 2025 Jun 17;16:1538938. doi: 10.3389/fphys.2025.1538938 (PMC12208852; doi:10.3389/fphys.2025.1538938)
Supplement: Supplementary file 1 [file DataSheet1.docx]

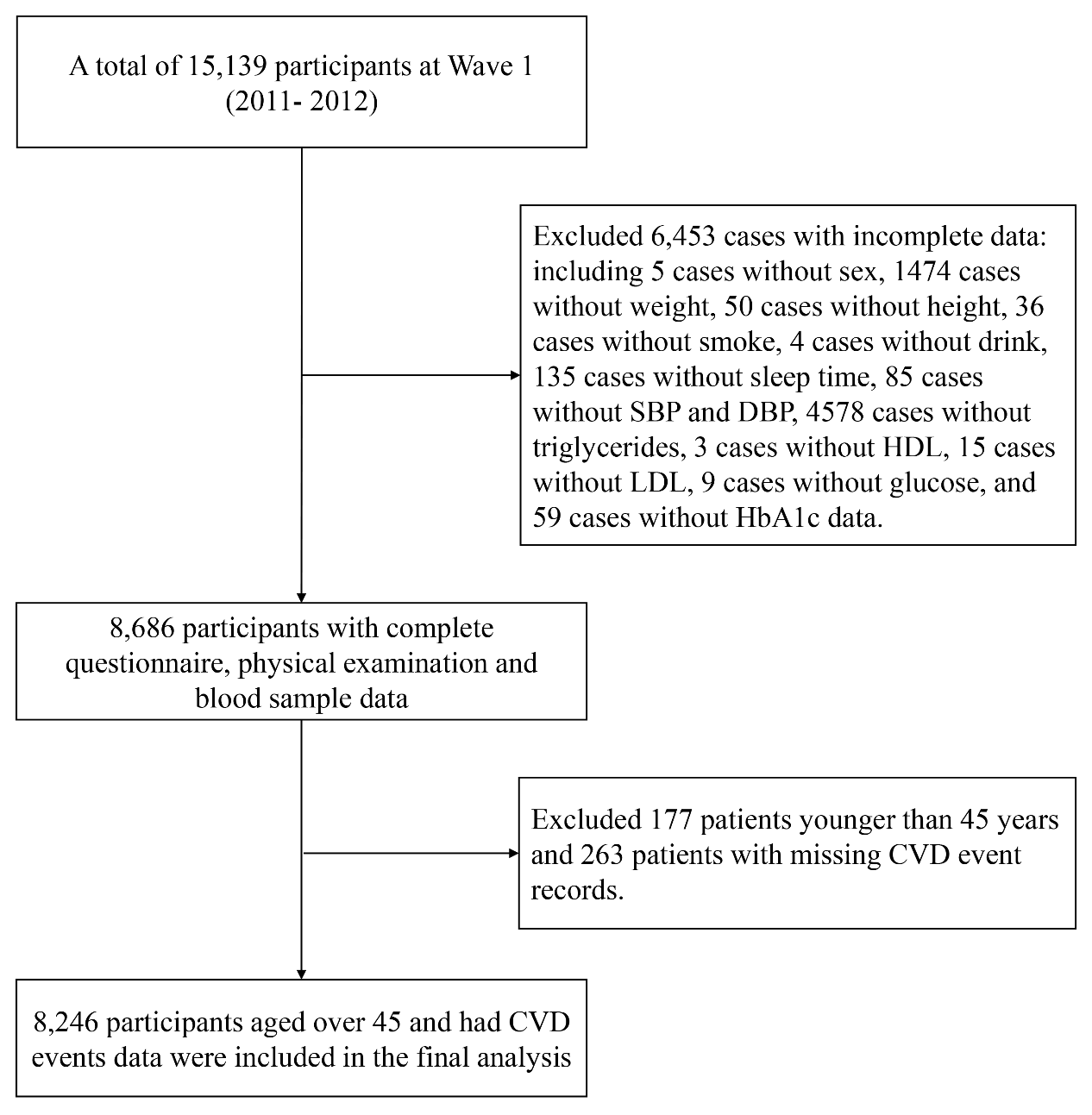


Figure S1 Flow diagram of the study population. CVD, cardiovascular disease; LDL-C, low-density lipoprotein-cholesterol; HDL-C, high-density lipoprotein-cholesterol; SBP, systolic blood pressure; DBP, diastolic blood pressure; HbA1c, hemoglobin A1c;


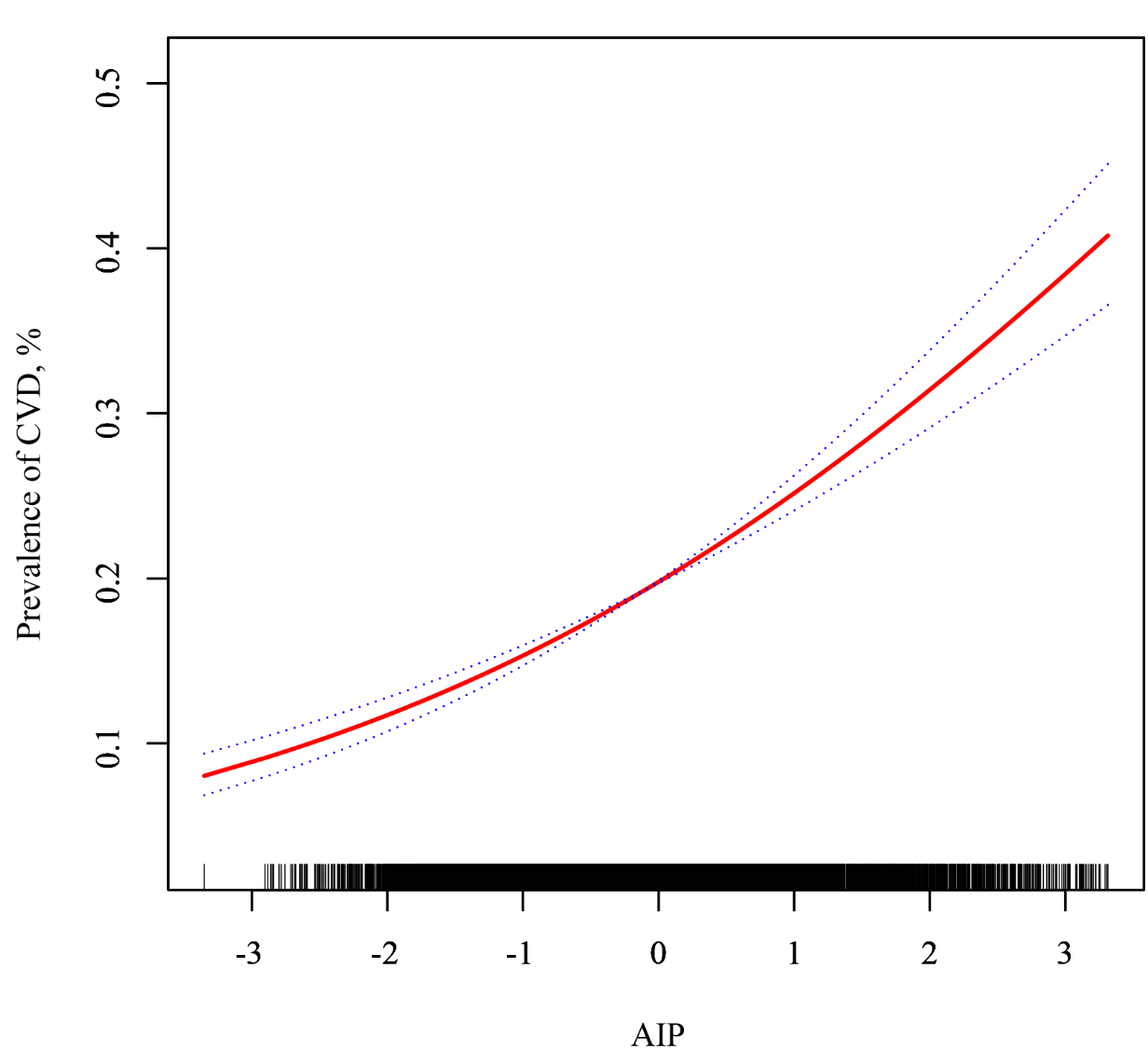


Figure. S2 The association between AIP and the risk of CVD.


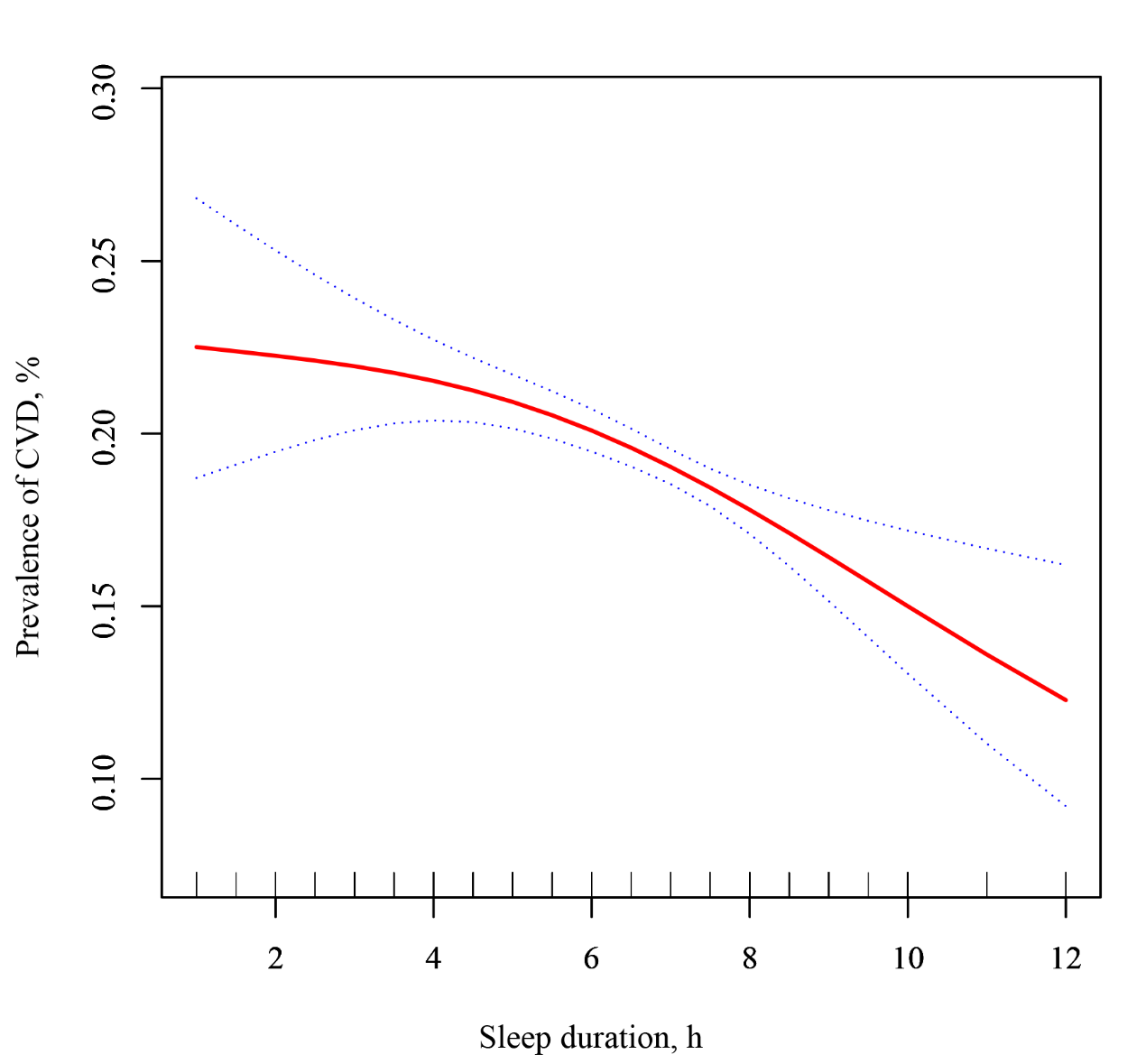


Figure. S3 The association between sleep duration and the risk of CVD.


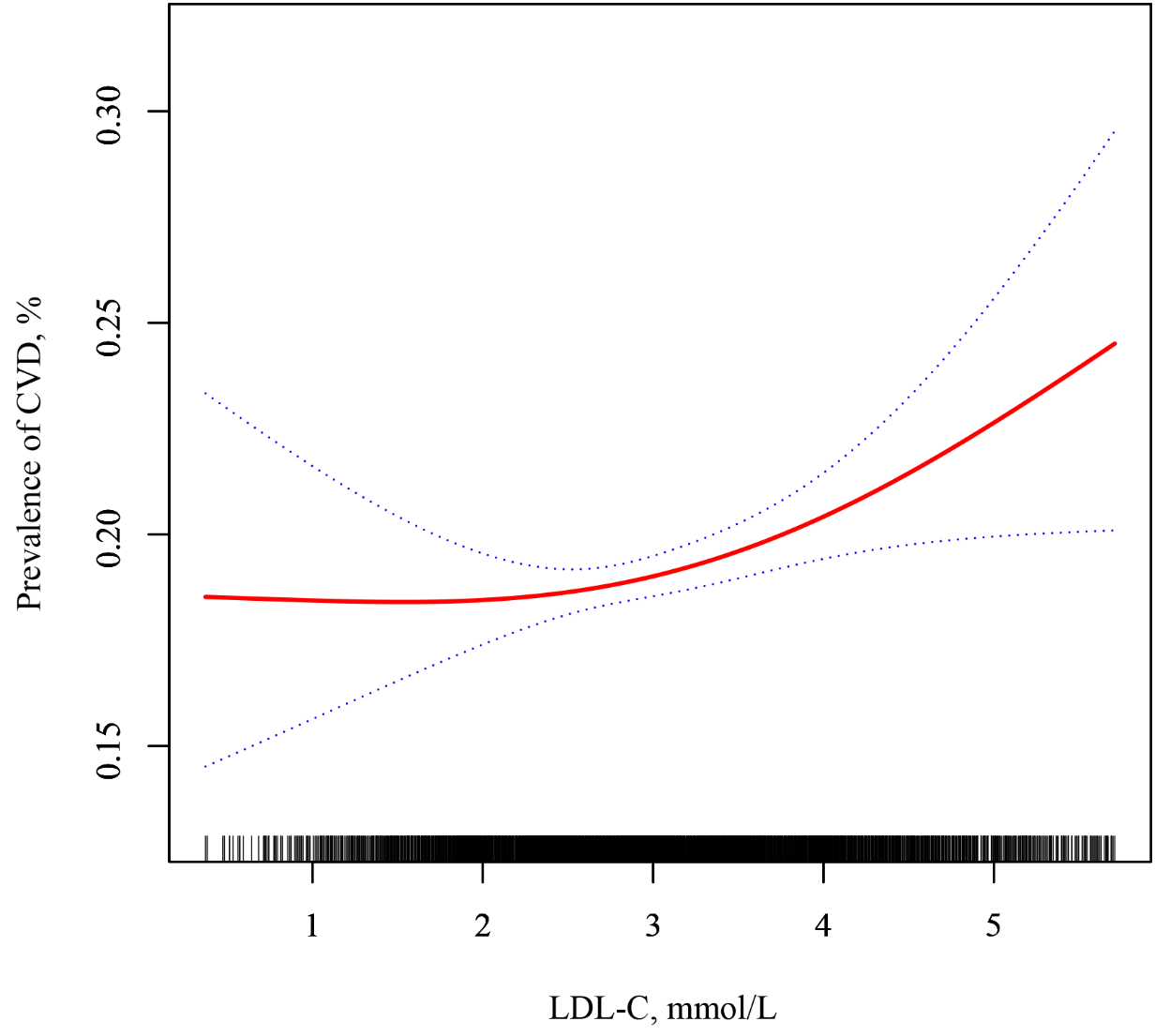


Figure. S4 The association between LDL-C and the risk of CVD.


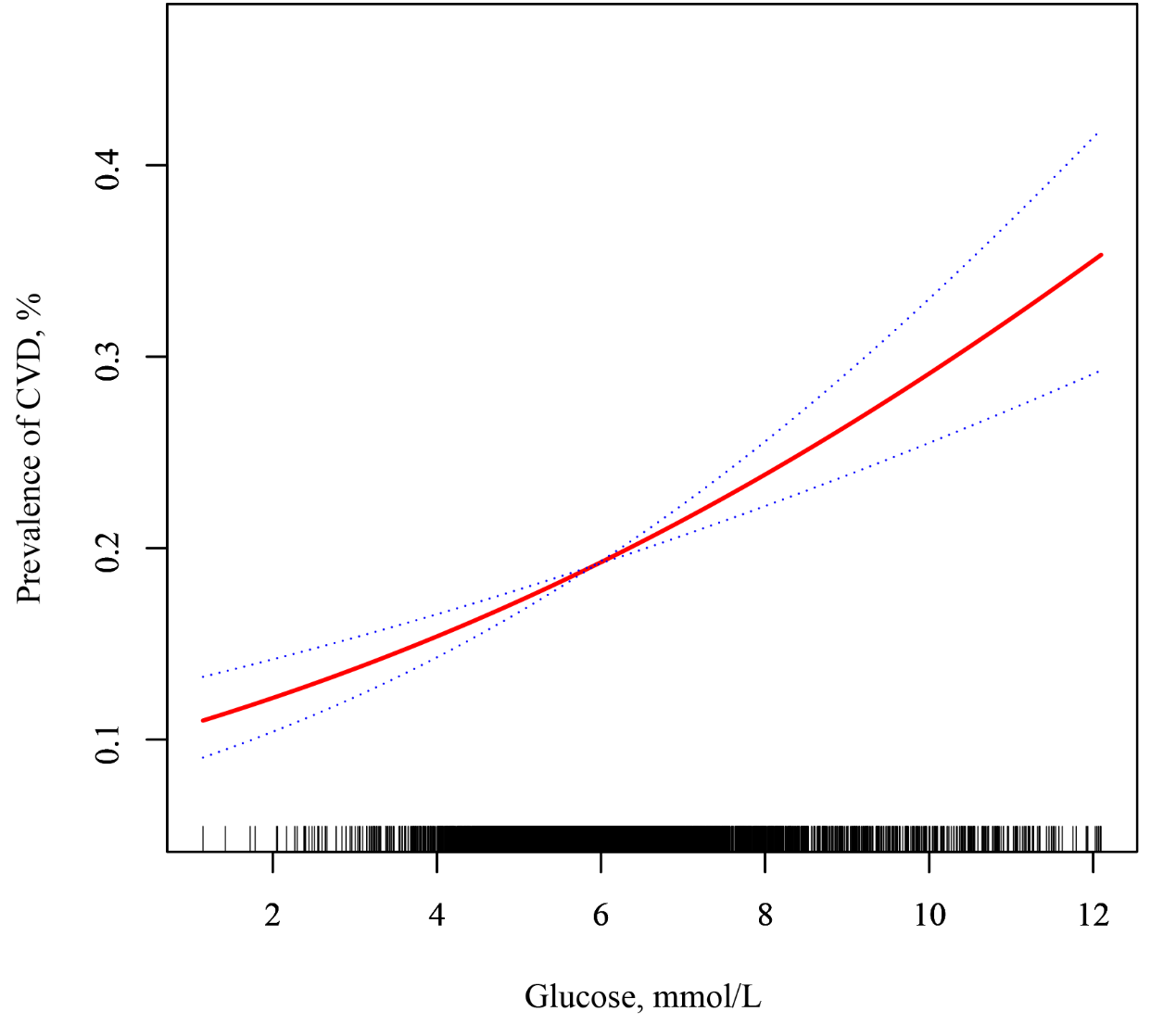


Figure. S5 The association between glucose and the risk of CVD.

| **Table S1.** Baseline characteristics stratified by sleep duration classification | | | | |
| --- | --- | --- | --- | --- |
|  | Sleep duration, h |  |  |  |
| Characteristics | <6 (n = 2457) | 6–8 (n = 3305) | ≥8 (n = 2484) | P-value |
| Age, years | 61.14 ± 9.25 | 58.37 ± 8.78 | 59.03 ± 9.46 | <0.001 |
| Sex, n (%) |  |  |  | <0.001 |
| Male | 1060 (43.14%) | 1585 (47.96%) | 1218 (49.03%) |  |
| Female | 1397 (56.86%) | 1720 (52.04%) | 1266 (50.97%) |  |
| Smoking status, n (%) | |  |  | 0.092 |
| Yes | 922 (37.53%) | 1326 (40.12%) | 995 (40.06%) |  |
| No | 1535 (62.47%) | 1979 (59.88%) | 1489 (59.94%) |  |
| Drinking status, n (%) | |  |  | 0.003 |
| None | 1713 (69.72%) | 2170 (65.66%) | 1648 (66.34%) |  |
| Mild or moderate | 744 (30.28%) | 1135 (34.34%) | 836 (33.66%) |  |
| BMI, n (%) |  |  |  | <0.001 |
| ≥24 kg/m2 | 860 (36.15%) | 1394 (43.55%) | 972 (40.33%) |  |
| <24 kg/m2 | 1519 (63.85%) | 1807 (56.45%) | 1438 (59.67%) |  |
| Physical activity, n (%) | |  |  | 0.035 |
| Inactive | 1531 (62.31%) | 2010 (60.82%) | 1502 (60.47%) |  |
| Insufficiently active | 846 (34.43%) | 1144 (34.61%) | 858 (34.54%) |  |
| Active | 80 (3.26%) | 151 (4.57%) | 124 (4.99%) |  |
| SBP, n (%) |  |  |  | 0.57 |
| ≥120 mmHg | 1588 (64.63%) | 2119 (64.11%) | 1626 (65.46%) |  |
| <120 mmHg | 869 (35.37%) | 1186 (35.89%) | 858 (34.54%) |  |
| DBP, n (%) |  |  |  | 0.013 |
| ≥80 mmHg | 786 (32.04%) | 1132 (34.32%) | 892 (36.00%) |  |
| <80 mmHg | 1667 (67.96%) | 2166 (65.68%) | 1586 (64.00%) |  |
| Hypertension, n (%) | 645 (26.31%) | 784 (23.74%) | 581 (23.42%) | 0.032 |
| Diabetes, n (%) | 157 (6.42%) | 204 (6.19%) | 115 (4.64%) | 0.012 |
| Kidney disease, n (%) | 214 (8.74%) | 192 (5.83%) | 132 (5.32%) | <0.001 |
| Hyperuricemia, n (%) | 113 (4.60%) | 167 (5.05%) | 115 (4.63%) | 0.658 |
| CVD, n (%) | 558 (22.71%) | 624 (18.88%) | 412 (16.59%) | <0.001 |
| LDL-C, n (%) |  |  |  | 0.087 |
| ≥3.12 mmol/L | 1080 (43.96%) | 1449 (43.84%) | 1025 (41.26%) |  |
| <3.12 mmol/L | 1377 (56.04%) | 1856 (56.16%) | 1459 (58.74%) |  |
| Glucose, n (%) |  |  |  | 0.124 |
| ≥6.1 mmol/L | 826 (33.62%) | 1059 (32.04%) | 768 (30.92%) |  |
| <6.1 mmol/L | 1631 (66.38%) | 2246 (67.96%) | 1716 (69.08%) |  |
| HbA1c, n (%) |  |  |  | 0.504 |
| ≥6 % | 184 (7.49%) | 270 (8.17%) | 207 (8.33%) |  |
| <6 % | 2273 (92.51%) | 3035 (91.83%) | 2277 (91.67%) |  |
| TG, mmol/L | 1.15 (0.83-1.64) | 1.16 (0.81-1.70) | 1.16 (0.82-1.70) | 0.353 |
| HDL-C, mmol/L | 1.37 ± 0.40 | 1.33 ± 0.39 | 1.32 ± 0.38 | <0.001 |
| LDL-C, mmol/L | 3.05 ± 0.91 | 3.05 ± 0.89 | 3.02 ± 0.89 | 0.329 |
| SUA, umol/L | 261.79 ± 72.94 | 263.76 ± 73.78 | 264.09 ± 73.58 | 0.484 |
| AIP | -0.13 ± 1.03 | -0.09 ± 1.08 | -0.06 ± 1.06 | 0.075 |

Data are shown as mean ± standard deviation (SD) or median (IQR) for continuous variables and proportions (%) for categorical variables.

CVD, cardiovascular diseases; BMI, body mass index; SBP, systolic blood pressure; DBP, diastolic blood pressure; LDL-C, low density lipoprotein cholesterol; HbA1c, glycosylated hemoglobin; TG, triglycerides; HDL-C, high density lipoprotein cholesterol; SUA, serum uric acid; AIP, atherogenic index of plasma.

| **Table S2.** Baseline characteristics stratified by LDL-C classification | | | |
| --- | --- | --- | --- |
|  | LDL-C, mmol/L |  |  |
| Characteristics | ≥3.12 (n = 3554) | <3.12 (n = 4692) | P-value |
| Age, years | 60.02 ± 9.00 | 58.92 ± 9.32 | <0.001 |
| Sex, n (%) |  |  | <0.001 |
| Male | 1455 (40.94%) | 2408 (51.32%) |  |
| Female | 2099 (59.06%) | 2284 (48.68%) |  |
| Smoking status, n (%) | |  | <0.001 |
| Yes | 1252 (35.23%) | 1991 (42.43%) |  |
| No | 2302 (64.77%) | 2701 (57.57%) |  |
| Drinking status, n (%) | |  | <0.001 |
| None | 2475 (69.64%) | 3056 (65.13%) |  |
| Mild or moderate | 1079 (30.36%) | 1636 (34.87%) |  |
| BMI, n (%) |  |  | <0.001 |
| ≥24 kg/m2 | 1574 (45.81%) | 1652 (36.28%) |  |
| <24 kg/m2 | 1862 (54.19%) | 2902 (63.72%) |  |
| Physical activity, n (%) | |  | 0.293 |
| Inactive | 2168 (61.00%) | 2875 (61.27%) |  |
| Insufficiently active | 1246 (35.06%) | 1602 (34.14%) |  |
| Active | 140 (3.94%) | 215 (4.58%) |  |
| Sleep duration, n (%) | |  | 0.087 |
| <6 | 1080 (30.39%) | 1377 (29.35%) |  |
| 6–8 | 1449 (40.77%) | 1856 (39.56%) |  |
| ≥8 | 1025 (28.84%) | 1459 (31.10%) |  |
| SBP, n (%) |  |  | <0.001 |
| ≥120 mmHg | 2411 (67.84%) | 2922 (62.28%) |  |
| <120 mmHg | 1143 (32.16%) | 1770 (37.72%) |  |
| DBP, n (%) |  |  | 0.021 |
| ≥80 mmHg | 1260 (35.53%) | 1550 (33.10%) |  |
| <80 mmHg | 2286 (64.47%) | 3133 (66.90%) |  |
| Hypertension, n (%) | 932 (26.25%) | 1078 (23.01%) | <0.001 |
| Diabetes, n (%) | 215 (6.08%) | 261 (5.57%) | 0.335 |
| Kidney disease, n (%) | 216 (6.09%) | 322 (6.88%) | 0.154 |
| Hyperuricemia, n (%) | 196 (5.51%) | 199 (4.24%) | 0.007 |
| CVD, n (%) | 748 (21.05%) | 846 (18.03%) | <0.001 |
| Glucose, n (%) | |  | 0.048 |
| ≥6.1 mmol/L | 1185 (33.34%) | 1468 (31.29%) |  |
| <6.1 mmol/L | 2369 (66.66%) | 3224 (68.71%) |  |
| HbA1c, n (%) | |  | <0.001 |
| ≥6 % | 345 (9.71%) | 316 (6.73%) |  |
| <6 % | 3209 (90.29%) | 4376 (93.27%) |  |
| TG, mmol/L | 1.23 (0.90-1.69) | 1.09 (0.76-1.66) | <0.001 |
| HDL-C, mmol/L | 1.36 ± 0.36 | 1.32 ± 0.42 | <0.001 |
| LDL-C, mmol/L | 3.84 ± 0.63 | 2.43 ± 0.49 | <0.001 |
| SUA, umol/L | 266.74 ± 73.36 | 260.65 ± 73.44 | <0.001 |
| AIP | -0.07 ± 0.90 | -0.11 ± 1.16 | <0.001 |

Data are shown as mean ± standard deviation (SD) or median (IQR) for continuous variables and proportions (%) for categorical variables.

CVD, cardiovascular diseases; BMI, body mass index; SBP, systolic blood pressure; DBP, diastolic blood pressure; LDL-C, low density lipoprotein cholesterol; HbA1c, glycosylated hemoglobin; TG, triglycerides; HDL-C, high density lipoprotein cholesterol; SUA, serum uric acid; AIP, atherogenic index of plasma.

| **Table S3.** Baseline characteristics stratified by glucose classification | | | |
| --- | --- | --- | --- |
|  | Glucose, mmol/L | |  |
| Characteristics | ≥6.1 (n = 2653) | <6.1 (n = 5593) | P-value |
| Age, years | 60.37 ± 9.03 | 58.93 ± 9.24 | <0.001 |
| Sex, n (%) |  |  | 0.274 |
| Male | 1266 (47.72%) | 2597 (46.43%) | |
| Female | 1387 (52.28%) | 2996 (53.57%) | |
| Smoking status, n (%) | |  | 0.062 |
| Yes | 1082 (40.78%) | 2161 (38.64%) | |
| No | 1571 (59.22%) | 3432 (61.36%) | |
| Drinking status, n (%) | |  | 0.467 |
| None | 1765 (66.53%) | 3766 (67.33%) | |
| Mild or moderate | 888 (33.47%) | 1827 (32.67%) | |
| BMI, n (%) |  |  | <0.001 |
| ≥24 kg/m2 | 1239 (48.25%) | 1987 (36.65%) | |
| <24 kg/m2 | 1329 (51.75%) | 3435 (63.35%) | |
| Physical activity, n (%) | |  | 0.502 |
| Inactive | 1638 (61.74%) | 3405 (60.88%) | |
| Insufficiently active | 910 (34.30%) | 1938 (34.65%) | |
| Active | 105 (3.96%) | 250 (4.47%) | |
| Sleep duration, n (%) | |  | 0.124 |
| <6 | 826 (31.13%) | 1631 (29.16%) | |
| 6–8 | 1059 (39.92%) | 2246 (40.16%) | |
| ≥8 | 768 (28.95%) | 1716 (30.68%) | |
| SBP, n (%) |  |  | <0.001 |
| ≥120 mmHg | 1941 (73.16%) | 3392 (60.65%) | |
| <120 mmHg | 712 (26.84%) | 2201 (39.35%) | |
| DBP, n (%) |  |  | <0.001 |
| ≥80 mmHg | 1006 (37.95%) | 1804 (32.34%) | |
| <80 mmHg | 1645 (62.05%) | 3774 (67.66%) | |
| Hypertension, n (%) | 807 (30.48%) | 1203 (21.53%) | <0.001 |
| Diabetes, n (%) | 334 (12.65%) | 142 (2.54%) | <0.001 |
| Kidney disease, n (%) | 154 (5.82%) | 384 (6.88%) | 0.069 |
| Hyperuricemia, n (%) | 180 (6.78%) | 215 (3.84%) | <0.001 |
| CVD, n (%) | 613 (23.11%) | 981 (17.54%) | <0.001 |
| LDL-C, n (%) | |  | 0.048 |
| ≥3.12 mmol/L | 1185 (44.67%) | 2369 (42.36%) | |
| <3.12 mmol/L | 1468 (55.33%) | 3224 (57.64%) | |
| HbA1c, n (%) | |  | <0.001 |
| ≥6 % | 537 (20.24%) | 124 (2.22%) | |
| <6 % | 2116 (79.76%) | 5469 (97.78%) | |
| TG, mmol/L | 1.41 (0.94-2.18) | 1.07 (0.78-1.51) | <0.001 |
| HDL-C, mmol/L | 1.27 ± 0.41 | 1.37 ± 0.38 | <0.001 |
| LDL-C, mmol/L | 3.06 ± 0.98 | 3.03 ± 0.85 | 0.083 |
| SUA, umol/L | 272.36 ± 77.11 | 258.97 ± 71.28 | <0.001 |
| AIP | 0.27 ± 1.18 | -0.26 ± 0.95 | <0.001 |

Data are shown as mean ± standard deviation (SD) or median (IQR) for continuous variables and proportions (%) for categorical variables.

CVD, cardiovascular diseases; BMI, body mass index; SBP, systolic blood pressure; DBP, diastolic blood pressure; LDL-C, low density lipoprotein cholesterol; HbA1c, glycosylated hemoglobin; TG, triglycerides; HDL-C, high density lipoprotein cholesterol; SUA, serum uric acid; AIP, atherogenic index of plasma.
